# Response to Drs. Anand, Craig, and Williams

**DOI:** 10.1097/PR9.0000000000000812

**Published:** 2020-03-25

**Authors:** Murat Aydede

**Affiliations:** Department of Philosophy, University of British Columbia, Vancouver, BC, Canada

Drs. Anand, Williams, and Craig (A&W&C) accuse me of using words in my original article^3^ that, according to them, constitute an unwarranted attack on their personal integrity. They also extend their accusation to the editor and the senior editorial board of *PAIN Reports* because they have apparently tolerated such an attack. I'm puzzled how they've arrived at this idea. I am worried that they have not understood my criticism and the reasoning behind it, focusing instead on the alleged inappropriate use of words taken out of context.

Let me start by repeating what I said in my original article.^3^ I think that anybody who claims that the first clause of the current IASP definition requires an ability of linguistic self-report on the part of the pain subject would be making a patently absurd claim that would be uncharitable, and to that extent, unfair, to the original authors of the IASP definition of pain. The IASP definition goes like this: pain is (1) *“an unpleasant sensory and emotional experience associated with actual or potential tissue damage*, or (2) described in terms of such damage.” It is self-evident that (1) *on its own* doesn't require any ability of linguistic self-report. To think otherwise and attribute it to the authors of the IASP definition would therefore border on the intentional misrepresentation. Did I think that A&W&C had thought that (1) on its own requires the ability of self-report? No, of course not. Is there anyone who thinks that? None as far as I know—and for good reasons. So, of course, A&W&C don't think that (1) requires self-report. Note Williams & Craig's own proposal for updating the definition of pain: “Pain is a distressing experience associated with actual or potential tissue damage with sensory, emotional, cognitive, and social components.”^4^ If Williams and Craig think their own proposed definition doesn't require the ability of linguistic self-report, they clearly must think that (1) by itself doesn't either. So, obviously, I am not making any claim about *their making* a patently absurd, uncharitable, and unfair claim. As far as I know, nobody has actually made any such claim, nor have I claimed otherwise. Therefore, I don't understand the *basis* for their accusation that I'm attacking their personal integrity. I stand by my wording in my original article, and invite them to pay closer attention to what I actually wrote and how the argument goes.

Anand and Craig's main claim was that the IASP definition itself as a whole “does not apply to living organisms that are incapable of self-report.”^1^ How did they arrive at this? Well, not because they think that (1) by itself requires self-report, but that the *whole* definition does, because the second clause contains the word “described” which, they think, requires the ability of linguistic self-report. Alas, in their response, A&W&C ignore my main criticism, which was that they are confusing the logic of an or-statement (a disjunction) with the logic of an and-statement (a conjunction). In their response, they continue to make this very same mistake. My criticism was that the use of “described” in the second clause, (2) above, doesn't show that the *whole* IASP definition (which is a disjunction) requires self-report *as they claim*.

Furthermore, as I explained in my article, the use of “described,” even in the second clause, doesn't imply an ability of self-report. I've explained the rationale for the need to add the second clause. The use of “described” was a proxy for *resemblance*, and as such doesn't have much to do with linguistic abilities, let alone *self*-report. The draft proposal of the IASP Task Force, therefore, improves on the official IASP formulation by making the original intent of the definition explicit and more precise—just as my own reformulation does (I encourage interested readers to also read my longer and more scholarly discussion of the IASP definition in its historic and intellectual context in my 2017 article.^2^)

A&C&W say that, in a definition of pain, “a narrow focus on sensory and affective features of the experience may lead to an overreliance on biomedical interventions (pharmaceutical, surgical, etc.), without giving due consideration to cognitive and social features of the pain experience or directing attention to psychological interventions and the social realities of people's lives, interventions consistent with the biopsychosocial model of pain.” The concern about overreliance on biomedical interventions at the expense of psychosocial interventions is quite legitimate, but addressing this concern by attempting to change the taxonomic definition of pain is misguided. It is also ill-advised to claim that all actual token pains (from trivial acute pains to severe chronic pains) are, by their essential nature, social. What is social about a trivial pain, say, due to an insect bite during my solo hiking? (And what does it mean to say this pain is social, actually?—see also Refs. 3,^5^) The majority of token pains that have ever occurred and will ever occur are of trivial acute pain variety that have no clinical significance. If we interpret “social” so loosely that each and every actual pain that has ever occurred and will ever occur in the history of animal kind (including humans) turns out to be social under that interpretation, then we risk trivializing sociality in clinically important cases where we have a much better grip on how social factors play a role in the development and treatment of problematic chronic pain. So, it's misguided to address A&C&W's concern by attempting to pack sociality into the taxonomic definition of pain that is supposed to collect *all* and *only* pains. The proper thing to do to address their concern is to continue to do good research that will contribute to our understanding of the biopsychosocial dynamics of chronic pain and rigorously deploy the results in clinical settings, and educate people. Let's not distort what a taxonomic definition is supposed to accomplish in our attempt to understand what makes *any* pain *pain*.

In their response, A&W&C simply repeat their original criticisms without engaging with the substance of the arguments I gave for my negative evaluations of their criticisms in my article. About half of their response concerns how the editorial board failed in their responsibility to maintain civilized discourse due to my alleged attacks on their personal integrity. It is telling that their judgment is based on a superficial reading of what I actually said or implied. I'll have to leave it to the readers to see for themselves whether, in their response, A&W&C correctly represent my views or adequately address my criticisms, and who is in fact transgressing the civil discourse in scholarly communications by asking the editor to press for retraction.

Finally, let me take this occasion to express my wish not to get bogged down in a debate that I consider to be unproductive at this point. Rather, I would like to see more positive discussion of the ideas I've floated in the last part of my original article prompted by Howard Fields' insightful criticism of the IASP definition. Some forms of nociplastic and neuropathic pains *may* pose difficulties for any definition of pain along the lines of the reformulation I've proposed in my article.^3^ These difficulties, if they turn out to be genuine difficulties indeed, apply to the draft formulation of the Task Force as well. I've discussed these issues in my article, but I hope that more critical and constructive thinking about these questions is yet to come.

## Disclosures

The author has no conflicts of interest to declare.
